# Dibromido[1,1′-dibutyl-2,2′-(pentane-1,1-di­yl)di-1*H*-benzimidazole]­copper(II)

**DOI:** 10.1107/S160053681002088X

**Published:** 2010-06-16

**Authors:** Robert T. Stibrany, Joseph A. Potenza

**Affiliations:** a Department of Chemistry and Chemical Biology, Rutgers, The State University of New Jersey, 610 Taylor Road, Piscataway, New Jersey 08854, USA

## Abstract

In the title compound, [CuBr_2_(C_27_H_36_N_4_)], the Cu^II^ ion exhibits a distorted tetra­hedral coordination geometry provided by two bromide ions and by chelation of two imine N-atom donors from a bis­(benzimidazole) ligand. Chelation results in a six-membered boat-shaped ring which links the benzimidazole groups. Each bis­(benzimidazole) fragment contains three *n*-butyl substituents, two of which have the expected *trans* conformation; the third exhibits the higher-energy *cis* conformation, an orientation consistent with several short intra­molecular C—H⋯Br inter­actions. Essentially planar (r.m.s. deviations of 0.0101 and 0.0183 Å) benzimidazole groups are oriented so as to give the bis­(benzimidazole) fragment a V-shaped appearance in profile with the *cis* and *trans n*-butyl groups directed to opposite sides of the planes. In the crystal, columns of mol­ecules along the *b*-axis direction form layers parallel to the (202) planes. Within a given column, the mol­ecules are linked by C—H⋯Br hydrogen bonds. The mol­ecules in adjacent columns are also linked by inter­molecular C—H⋯π interactions, forming a three-dimensional network.

## Related literature

For the applications of bis­(imidazoles), bis­(benzimidazoles), and their complexes with metal ions, see: Stibrany *et al.* (2002 ▶, 2003 ▶, 2004 ▶); Knapp *et al.* (1990 ▶). For related structures see: Stibrany (2009 ▶); Stibrany *et al.* 2005 ▶); Stibrany & Potenza (2006 ▶, 2008 ▶); Hou *et al.* (2006 ▶).
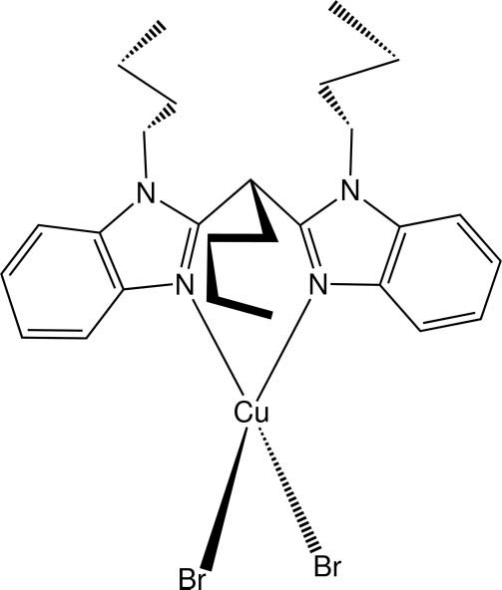


## Experimental

### 

#### Crystal data

[CuBr_2_(C_27_H_36_N_4_)]
*M* *_r_* = 639.96
Monoclinic, 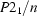

*a* = 13.521 (2) Å
*b* = 14.604 (3) Å
*c* = 13.881 (2) Å
β = 96.636 (3)°
*V* = 2722.6 (8) Å^3^
*Z* = 4
Mo *K*α radiation
μ = 3.76 mm^−1^
*T* = 100 K
0.45 × 0.18 × 0.07 mm

#### Data collection

Bruker SMART CCD area-detector diffractometer
Absorption correction: multi-scan (*SADABS*; Bruker, 2000 ▶) *T*_min_ = 0.644, *T*_max_ = 1.00
25644 measured reflections
5405 independent reflections
4692 reflections with *I* > 2σ(*I*)
*R*_int_ = 0.038

#### Refinement

*R*[*F* ^2^ > 2σ(*F* ^2^)] = 0.027
*wR*(*F* ^2^) = 0.066
*S* = 1.00
5405 reflections
310 parameters
H-atom parameters constrained
Δρ_max_ = 0.74 e Å^−3^
Δρ_min_ = −0.38 e Å^−3^

### 

Data collection: *SMART* (Bruker, 2000 ▶); cell refinement: *SAINT-Plus* (Bruker, 2000 ▶); data reduction: *SAINT-Plus*; program(s) used to solve structure: *SHELXS97* (Sheldrick, 2008 ▶); program(s) used to refine structure: *SHELXL97* (Sheldrick, 2008 ▶); molecular graphics: *ORTEPIII* (Burnett & Johnson, 1996 ▶) and *ORTEP-32* (Farrugia, 1997 ▶); software used to prepare material for publication: *SHELXTL* (Sheldrick, 2008 ▶).

## Supplementary Material

Crystal structure: contains datablocks I, global. DOI: 10.1107/S160053681002088X/lh5058sup1.cif

Structure factors: contains datablocks I. DOI: 10.1107/S160053681002088X/lh5058Isup2.hkl

Additional supplementary materials:  crystallographic information; 3D view; checkCIF report

## Figures and Tables

**Table:** 

Cu1—N23	1.9536 (19)
Cu1—N13	1.994 (2)
Cu1—Br1	2.3563 (5)
Cu1—Br2	2.3608 (5)

**Table:** 

N23—Cu1—N13	90.44 (8)
N23—Cu1—Br1	130.64 (6)
N13—Cu1—Br1	106.87 (6)
N23—Cu1—Br2	98.49 (6)
N13—Cu1—Br2	134.58 (6)
Br1—Cu1—Br2	100.523 (16)
C22—C1—C12	110.63 (19)

**Table 2:** Hydrogen-bond geometry (Å, °) *Cg*1 is the centroid of the N11/C11/C13/N13/C12 ring.

*D*—H⋯*A*	*D*—H	H⋯*A*	*D*⋯*A*	*D*—H⋯*A*
C14—H14⋯Br1	0.95	2.79	3.551 (3)	138
C17—H17⋯Br2^i^	0.95	2.90	3.606 (3)	132
C18—H18*A*⋯Br1^ii^	0.99	2.86	3.741 (3)	148
C5—H5*B*⋯*Cg*1^ii^	0.98	2.87	3.631 (3)	135
C2*B*—H2*B*1⋯*Cg*1^iii^	0.98	2.82	3.777 (3)	165

Symmetry codes: (i) 
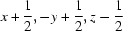
; (ii) 
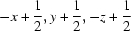
; (iii) 
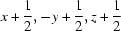
.

**Fig. 1.**
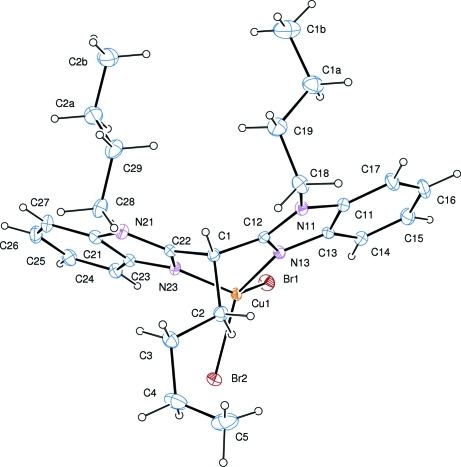
The molecular structure of the title compound (I) showing the atom-numbering scheme. Displacement ellipsoids are shown at the 50% probability level. H atoms are shown as spheres of arbitrary radius.

**Fig. 2.**
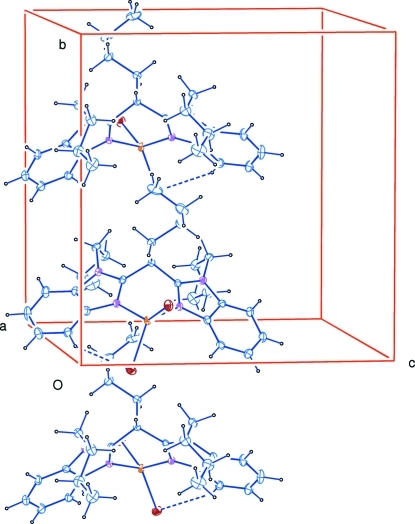
View showing columns of the core structure of (I) along the b axis direction. H atoms and 1-butyl groups have been omitted for clarity.

**Fig. 3.**
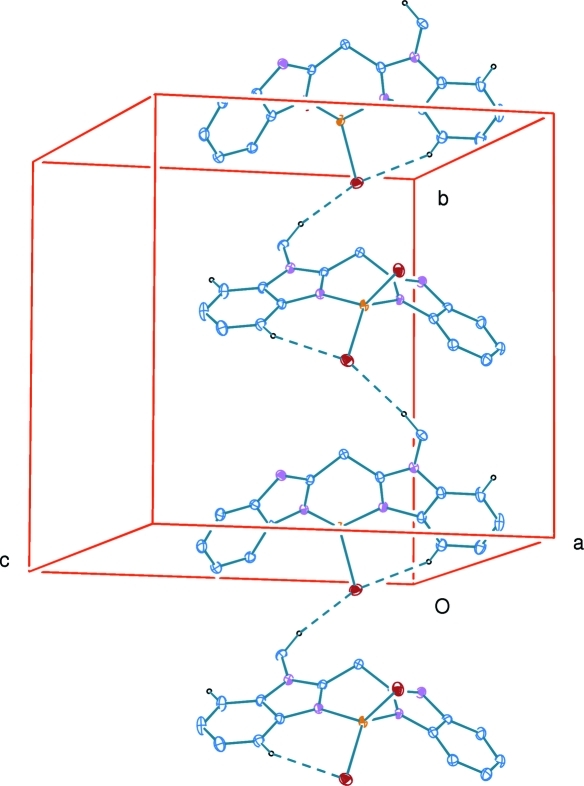
View, showing a column of molecules of (I) extending along the b axis direction.

**Fig. 4.**
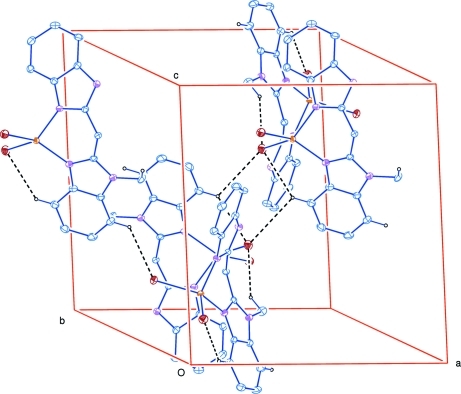
View, approximately along the b axis direction showing the C—H···Br hydrogen bonds which link the columns shown in Fig. 2. Except for those involved in hydrogen bonding, H atoms and 1-butyl C atoms have been omitted for clarity.

## supplementary crystallographic information

### Comment

The title compound (I) was prepared as part of our long-term interest in the chemistry of bis(imidazoles), bis(benzimidazoles), and their complexes with metal ions. These species have demonstrated their usefulness as proton sponges (Stibrany *et al.*, 2002), geometrically constraining ligands (Stibrany *et al.*, 2004), agents to study electron transfer (Knapp *et al.*, 1990), polymerization catalysts (Stibrany *et al.*, 2003), and in the formation of metal-organic copolymers (Stibrany & Potenza, 2008).

The structure of [1,1'-bis(1-butylbenzimidazol-2-yl) pentane]copper(II) dibromide, (I), contains molecules (Fig. 1) in which two essentially planar benzimidazole fragments are linked by the a bridging (bridgehead) carbon atom C1 and a Cu(II) ion, which forms Cu—*N*(imine) bonds to N13 and N23, to complete a six-membered Cu1—N13—C12—C1—C22—N23- ring. The ring adopts a boat conformation with the copper(II) ion and the bridgehead carbon atom corresponding to the bow and stern, respectively. The angles N23—Cu1—N13 and C22—C1—C12 (Table 1), at the bow and stern, respectively, give the molecule a V-shape in profile (Fig. 2). Two bromine atoms, Br1 and Br2, complete a distorted-teterrahedral coordination geometry at Cu1, as evidenced by the several angles at Cu1 (Table 1) and by the "tetrahedral twist dihedral angle" N13—Cu1—N23/Br1—Cu1—Br2, 65.08 (6)°. Of the three *n*-butyl groups, two exhibit the *trans* conformation and extend above the planes of the benzimidazole fragments (Fig. 1), while the third, bonded to the bridgehead carbon atom C1, exhibits the higher-energy *cis *conformation and is positioned below the planes of the benzimidazole rings. The *cis* orientation is consistent with several intramolecular C—H···Br interactions whose H···Br (Br2···H2B, 3.1147 Å and Br2···H4A, 3.6145 Å) distances are too long to be considered hydrogen bonds, yet too short to be ignored. Lastly, we note that the complex exhibits an intramolecular C14—H14···Br1 hydrogen bond (Table 2).

In the crystal, molecules of (I) form columns along the b cell direction (Fig. 2) centered about the twofold screw axes at 1/4 b 1/4 and symmetry related positions in space group *P*2_1_/*n*. Within a given column, the molecules are linked by C18—H18*b*···Br1 hydrogen bonds (Fig. 3) to give each column spirial staircase appearance along its length. The columns are arranged in layers parallel to the (2 0 2) planes (Fig.2), and are linked together by intermolecular C17—H17···Br2 hydrogen bonds (Fig. 4) to yield a three-dimensional network structure. The C—H and H···Br distances for the C—H···Br hydrogen bonds in (I) (Table 2) compare favorably with those reported previously for a distorted-tetrahedral Cu(I) bromide complex (Hou *et al.*, 2006).

In related structures, alkyl chains, substituted at the *N*(amine) and bridgehead positions of bis(benzimidazoles), have been observed in three permutations with respect to the benzimidazole planes: all to one side, two up, bridgehead substituent down as in the present instance, and two up, *N*(amine) substituent down (Stibrany, 2009). In the structure of the free ligand of (I) (Stibrany *et al.*, 2003), all three alkyl chains assume the *trans* conformation. Presumably, the way in which these molecules pack in a crystal determines to some extent the conformation of these substituents, or *vice versa. *In the analogous dichloride complex, the alkyl chains are arranged similarly to those in (I) (Stibrany *et al.*, 2003). In fact, (I) and its dichloro analogue are isomorphous.

### Experimental

Compound (I) was prepared from the addition of 200 mg (0.48 mmol) of [1,1'-bis(1-butylbenzimidazol-2-yl) pentane] (Stibrany *et al.*, 2003) and 107 mg (0.48 mmol) of CuBr_2_ to a mixture of 20 ml of ethanol and 2 ml of triethylorthoformate. This mixture was warmed gently for 5 min and then allowed to evaporate slowly. When the volume was reduced by approximately 60%, dark red crystals of (I) had formed and were collected by filtration, and dried in air. Yield 301 mg (yield 98.0%). (m.p. 486 K(melt) IR (KBr pellet, cm^-1^): 2957 (*s*), 2930 (*m*), 22871 (w), 1613 (w), 1509 (*m*), 1455 (*s*), 1281 (w), 1015 (w), 755 (*s*).

### Refinement

Hydrogen atoms were positioned geometrically using a riding model, with C—H = 0.95 and 1.00 Å, respectively, for *n*-butyl and benzimidazole H atom, and *U*_iso_(H) = 1.2-1.5 *U*_eq _(C).

### Crystal data

**Table:**

[CuBr_2_(C_27_H_36_N_4_)]	*F*(000) = 1300
*M**_r_* = 639.96	*D*_x_ = 1.561 Mg m^−^^3^
Monoclinic, *P*2_1_/*n*	Melting point: 486 K
Hall symbol: -P 2yn	Mo *K*α radiation, λ = 0.71073 Å
*a* = 13.521 (2) Å	Cell parameters from 982 reflections
*b* = 14.604 (3) Å	θ = 2.2–25.9°
*c* = 13.881 (2) Å	µ = 3.76 mm^−^^1^
β = 96.636 (3)°	*T* = 100 K
*V* = 2722.6 (8) Å^3^	Blade, red
*Z* = 4	0.45 × 0.18 × 0.07 mm

### Data collection

**Table:**

Bruker SMART CCD area-detector diffractometer	5405 independent reflections
Radiation source: fine-focus sealed tube	4692 reflections with *I* > 2σ(*I*)
graphite	*R*_int_ = 0.038
φ and ω scans	θ_max_ = 26.1°, θ_min_ = 2.0°
Absorption correction: multi-scan (*SADABS*; Bruker, 2000)	*h* = −16→16
*T*_min_ = 0.644, *T*_max_ = 1.00	*k* = −18→18
25644 measured reflections	*l* = −17→16

### Refinement

**Table:**

Refinement on *F*^2^	Primary atom site location: structure-invariant direct methods
Least-squares matrix: full	Secondary atom site location: difference Fourier map
*R*[*F*^2^ > 2σ(*F*^2^)] = 0.027	Hydrogen site location: inferred from neighbouring sites
*wR*(*F*^2^) = 0.066	H-atom parameters constrained
*S* = 1.00	*w* = 1/[σ^2^(*F*_o_^2^) + (0.0324*P*)^2^ + 2.745*P*] where *P* = (*F*_o_^2^ + 2*F*_c_^2^)/3
5405 reflections	(Δ/σ)_max_ = 0.001
310 parameters	Δρ_max_ = 0.74 e Å^−^^3^
0 restraints	Δρ_min_ = −0.38 e Å^−^^3^

### Special details

**Table:**

Geometry. All e.s.d.'s (except the e.s.d. in the dihedral angle between two l.s. planes) are estimated using the full covariance matrix. The cell e.s.d.'s are taken into account individually in the estimation of e.s.d.'s in distances, angles and torsion angles; correlations between e.s.d.'s in cell parameters are only used when they are defined by crystal symmetry. An approximate (isotropic) treatment of cell e.s.d.'s is used for estimating e.s.d.'s involving l.s. planes.
Refinement. Refinement of *F*^2^ against ALL reflections. The weighted *R*-factor *wR* and goodness of fit *S* are based on *F*^2^, conventional *R*-factors *R* are based on *F*, with *F* set to zero for negative *F*^2^. The threshold expression of *F*^2^ > σ(*F*^2^) is used only for calculating *R*-factors(gt) *etc*. and is not relevant to the choice of reflections for refinement. *R*-factors based on *F*^2^ are statistically about twice as large as those based on *F*, and *R*- factors based on ALL data will be even larger.

### Fractional atomic coordinates and isotropic or equivalent isotropic displacement parameters (Å^2^)

**Table:**

	*x*	*y*	*z*	*U*_iso_*/*U*_eq_	
Cu1	0.10186 (2)	0.119645 (19)	0.230340 (19)	0.01124 (8)	
Br1	0.045118 (19)	−0.023221 (16)	0.167330 (18)	0.01896 (7)	
Br2	−0.049244 (18)	0.184464 (17)	0.267178 (17)	0.01820 (7)	
N11	0.34142 (15)	0.24098 (13)	0.12023 (14)	0.0130 (4)	
N13	0.20969 (15)	0.16010 (13)	0.15332 (14)	0.0122 (4)	
N21	0.30460 (14)	0.21513 (13)	0.45058 (14)	0.0131 (4)	
N23	0.18474 (14)	0.15045 (13)	0.35070 (13)	0.0115 (4)	
C1	0.27426 (17)	0.27602 (15)	0.27828 (16)	0.0116 (5)	
H1	0.3394	0.3081	0.2948	0.014*	
C2	0.18961 (18)	0.34819 (16)	0.26140 (17)	0.0146 (5)	
H2A	0.2043	0.3897	0.2085	0.018*	
H2B	0.1265	0.3162	0.2395	0.018*	
C3	0.17497 (19)	0.40545 (17)	0.35029 (18)	0.0188 (5)	
H3A	0.2319	0.4479	0.3640	0.023*	
H3B	0.1739	0.3646	0.4071	0.023*	
C4	0.0784 (2)	0.46057 (19)	0.3362 (2)	0.0280 (6)	
H4A	0.0215	0.4175	0.3301	0.034*	
H4B	0.0739	0.4983	0.3948	0.034*	
C5	0.0688 (2)	0.52288 (19)	0.2482 (2)	0.0321 (7)	
H5A	0.0632	0.4857	0.1890	0.048*	
H5B	0.1279	0.5621	0.2503	0.048*	
H5C	0.0093	0.5611	0.2484	0.048*	
C11	0.31456 (18)	0.18592 (16)	0.03975 (17)	0.0141 (5)	
C12	0.27675 (17)	0.22314 (16)	0.18544 (16)	0.0118 (5)	
C13	0.23188 (18)	0.13538 (16)	0.06078 (17)	0.0135 (5)	
C14	0.18405 (18)	0.07574 (17)	−0.00812 (17)	0.0159 (5)	
H14	0.1276	0.0411	0.0049	0.019*	
C15	0.22204 (19)	0.06923 (18)	−0.09575 (18)	0.0206 (5)	
H15	0.1911	0.0291	−0.1440	0.025*	
C16	0.3049 (2)	0.1202 (2)	−0.11563 (19)	0.0254 (6)	
H16	0.3288	0.1137	−0.1770	0.030*	
C17	0.3529 (2)	0.17963 (18)	−0.04871 (18)	0.0199 (5)	
H17	0.4091	0.2144	−0.0622	0.024*	
C18	0.42282 (19)	0.30825 (16)	0.12849 (19)	0.0175 (5)	
H18A	0.4043	0.3605	0.1682	0.021*	
H18B	0.4310	0.3319	0.0630	0.021*	
C19	0.52200 (19)	0.26958 (18)	0.17390 (19)	0.0211 (5)	
H19A	0.5114	0.2389	0.2355	0.025*	
H19B	0.5685	0.3212	0.1900	0.025*	
C1A	0.5710 (2)	0.20174 (19)	0.1109 (2)	0.0250 (6)	
H1A1	0.5247	0.1503	0.0936	0.030*	
H1A2	0.5842	0.2324	0.0501	0.030*	
C1B	0.6683 (2)	0.1643 (2)	0.1619 (2)	0.0350 (7)	
H1B1	0.6549	0.1296	0.2194	0.053*	
H1B2	0.7134	0.2151	0.1815	0.053*	
H1B3	0.6993	0.1239	0.1175	0.053*	
C21	0.26253 (18)	0.14778 (15)	0.50387 (17)	0.0131 (5)	
C22	0.25617 (17)	0.21300 (15)	0.35975 (16)	0.0112 (5)	
C23	0.18673 (18)	0.10799 (16)	0.44063 (17)	0.0129 (5)	
C24	0.12813 (18)	0.03726 (16)	0.47082 (17)	0.0152 (5)	
H24	0.0759	0.0107	0.4282	0.018*	
C25	0.14950 (19)	0.00760 (17)	0.56540 (18)	0.0187 (5)	
H25	0.1114	−0.0407	0.5884	0.022*	
C26	0.2264 (2)	0.04736 (17)	0.62821 (18)	0.0193 (5)	
H26	0.2392	0.0248	0.6926	0.023*	
C27	0.28417 (19)	0.11836 (17)	0.59950 (17)	0.0172 (5)	
H27	0.3357	0.1455	0.6425	0.021*	
C29	0.48784 (19)	0.24181 (18)	0.46606 (19)	0.0216 (6)	
H29A	0.5389	0.2877	0.4896	0.026*	
H29B	0.4872	0.2372	0.3948	0.026*	
C28	0.38695 (18)	0.27632 (17)	0.48784 (18)	0.0167 (5)	
H28A	0.3876	0.2827	0.5589	0.020*	
H28B	0.3751	0.3377	0.4586	0.020*	
C2A	0.5186 (2)	0.14970 (18)	0.5108 (2)	0.0226 (6)	
H2A1	0.4689	0.1029	0.4865	0.027*	
H2A2	0.5193	0.1536	0.5821	0.027*	
C2B	0.6213 (2)	0.1199 (2)	0.4870 (2)	0.0291 (6)	
H2B1	0.6709	0.1657	0.5114	0.044*	
H2B2	0.6203	0.1141	0.4166	0.044*	
H2B3	0.6384	0.0607	0.5177	0.044*	

### Atomic displacement parameters (Å^2^)

**Table:**

	*U*^11^	*U*^22^	*U*^33^	*U*^12^	*U*^13^	*U*^23^
Cu1	0.01029 (15)	0.01486 (14)	0.00862 (14)	−0.00107 (11)	0.00129 (11)	−0.00152 (11)
Br1	0.01931 (14)	0.01686 (13)	0.02065 (13)	−0.00290 (10)	0.00205 (10)	−0.00428 (10)
Br2	0.01408 (13)	0.02410 (14)	0.01700 (13)	0.00417 (10)	0.00434 (9)	−0.00078 (10)
N11	0.0141 (10)	0.0135 (10)	0.0115 (10)	−0.0003 (8)	0.0023 (8)	0.0012 (8)
N13	0.0134 (10)	0.0129 (9)	0.0106 (9)	0.0011 (8)	0.0025 (8)	−0.0001 (8)
N21	0.0134 (10)	0.0136 (10)	0.0121 (10)	−0.0023 (8)	0.0006 (8)	0.0010 (8)
N23	0.0133 (10)	0.0123 (9)	0.0088 (9)	0.0001 (8)	0.0007 (8)	0.0007 (7)
C1	0.0120 (11)	0.0123 (11)	0.0107 (11)	−0.0004 (9)	0.0019 (9)	0.0011 (9)
C2	0.0169 (13)	0.0129 (11)	0.0141 (12)	0.0003 (9)	0.0015 (9)	0.0011 (9)
C3	0.0214 (14)	0.0182 (12)	0.0172 (13)	0.0023 (10)	0.0038 (10)	−0.0035 (10)
C4	0.0233 (15)	0.0259 (14)	0.0359 (16)	0.0070 (12)	0.0078 (12)	−0.0091 (12)
C5	0.0291 (16)	0.0205 (14)	0.0444 (19)	0.0098 (12)	−0.0054 (14)	−0.0088 (13)
C11	0.0141 (12)	0.0166 (12)	0.0116 (11)	0.0024 (9)	0.0020 (9)	0.0021 (9)
C12	0.0108 (11)	0.0142 (11)	0.0104 (11)	0.0032 (9)	0.0011 (9)	0.0035 (9)
C13	0.0141 (12)	0.0156 (12)	0.0112 (11)	0.0057 (9)	0.0028 (9)	0.0011 (9)
C14	0.0155 (12)	0.0184 (12)	0.0136 (12)	0.0021 (10)	0.0005 (9)	−0.0002 (10)
C15	0.0188 (13)	0.0277 (14)	0.0148 (12)	−0.0010 (11)	−0.0005 (10)	−0.0048 (10)
C16	0.0232 (14)	0.0417 (16)	0.0121 (13)	0.0002 (12)	0.0063 (11)	−0.0020 (11)
C17	0.0174 (13)	0.0284 (14)	0.0151 (12)	−0.0025 (11)	0.0067 (10)	0.0003 (10)
C18	0.0197 (13)	0.0150 (12)	0.0192 (13)	−0.0050 (10)	0.0078 (10)	0.0023 (10)
C19	0.0161 (13)	0.0224 (13)	0.0247 (14)	−0.0059 (10)	0.0027 (11)	0.0011 (11)
C1A	0.0202 (14)	0.0263 (14)	0.0295 (15)	−0.0002 (11)	0.0073 (12)	0.0036 (12)
C1B	0.0213 (15)	0.0355 (17)	0.049 (2)	0.0031 (13)	0.0057 (14)	0.0073 (15)
C21	0.0140 (12)	0.0115 (11)	0.0142 (11)	−0.0002 (9)	0.0037 (9)	0.0002 (9)
C22	0.0114 (11)	0.0123 (11)	0.0103 (11)	0.0015 (9)	0.0025 (9)	−0.0009 (9)
C23	0.0128 (12)	0.0144 (11)	0.0117 (11)	0.0016 (9)	0.0019 (9)	−0.0013 (9)
C24	0.0150 (12)	0.0159 (12)	0.0147 (12)	−0.0028 (10)	0.0021 (9)	−0.0018 (9)
C25	0.0200 (13)	0.0190 (12)	0.0181 (13)	−0.0030 (10)	0.0068 (10)	0.0010 (10)
C26	0.0248 (14)	0.0213 (13)	0.0117 (12)	0.0016 (11)	0.0021 (10)	0.0012 (10)
C27	0.0211 (13)	0.0195 (12)	0.0104 (12)	−0.0013 (10)	−0.0004 (10)	−0.0002 (9)
C29	0.0164 (13)	0.0274 (14)	0.0201 (13)	−0.0068 (11)	−0.0017 (10)	0.0035 (11)
C28	0.0191 (13)	0.0158 (12)	0.0143 (12)	−0.0063 (10)	−0.0029 (10)	0.0010 (10)
C2A	0.0201 (14)	0.0242 (13)	0.0233 (14)	−0.0033 (11)	0.0017 (11)	−0.0019 (11)
C2B	0.0227 (15)	0.0338 (16)	0.0304 (16)	0.0012 (12)	0.0018 (12)	−0.0054 (13)

### Geometric parameters (Å, °)

**Table:**

Cu1—N23	1.9536 (19)	C16—C17	1.377 (4)
Cu1—N13	1.994 (2)	C16—H16	0.9500
Cu1—Br1	2.3563 (5)	C17—H17	0.9500
Cu1—Br2	2.3608 (5)	C18—C19	1.523 (4)
N11—C12	1.354 (3)	C18—H18A	0.9900
N11—C11	1.390 (3)	C18—H18B	0.9900
N11—C18	1.470 (3)	C19—C1A	1.523 (4)
N13—C12	1.332 (3)	C19—H19A	0.9900
N13—C13	1.400 (3)	C19—H19B	0.9900
N21—C22	1.352 (3)	C1A—C1B	1.522 (4)
N21—C21	1.391 (3)	C1A—H1A1	0.9900
N21—C28	1.474 (3)	C1A—H1A2	0.9900
N23—C22	1.325 (3)	C1B—H1B1	0.9800
N23—C23	1.391 (3)	C1B—H1B2	0.9800
C1—C22	1.500 (3)	C1B—H1B3	0.9800
C1—C12	1.506 (3)	C21—C27	1.394 (3)
C1—C2	1.554 (3)	C21—C23	1.397 (3)
C1—H1	1.0000	C23—C24	1.395 (3)
C2—C3	1.522 (3)	C24—C25	1.381 (3)
C2—H2A	0.9900	C24—H24	0.9500
C2—H2B	0.9900	C25—C26	1.403 (4)
C3—C4	1.527 (4)	C25—H25	0.9500
C3—H3A	0.9900	C26—C27	1.384 (4)
C3—H3B	0.9900	C26—H26	0.9500
C4—C5	1.517 (4)	C27—H27	0.9500
C4—H4A	0.9900	C29—C28	1.517 (4)
C4—H4B	0.9900	C29—C2A	1.519 (4)
C5—H5A	0.9800	C29—H29A	0.9900
C5—H5B	0.9800	C29—H29B	0.9900
C5—H5C	0.9800	C28—H28A	0.9900
C11—C17	1.390 (3)	C28—H28B	0.9900
C11—C13	1.398 (3)	C2A—C2B	1.526 (4)
C13—C14	1.396 (3)	C2A—H2A1	0.9900
C14—C15	1.377 (3)	C2A—H2A2	0.9900
C14—H14	0.9500	C2B—H2B1	0.9800
C15—C16	1.400 (4)	C2B—H2B2	0.9800
C15—H15	0.9500	C2B—H2B3	0.9800
			
N23—Cu1—N13	90.44 (8)	N11—C18—C19	113.6 (2)
N23—Cu1—Br1	130.64 (6)	N11—C18—H18A	108.9
N13—Cu1—Br1	106.87 (6)	C19—C18—H18A	108.9
N23—Cu1—Br2	98.49 (6)	N11—C18—H18B	108.9
N13—Cu1—Br2	134.58 (6)	C19—C18—H18B	108.9
Br1—Cu1—Br2	100.523 (16)	H18A—C18—H18B	107.7
C12—N11—C11	107.27 (19)	C18—C19—C1A	115.1 (2)
C12—N11—C18	127.7 (2)	C18—C19—H19A	108.5
C11—N11—C18	125.0 (2)	C1A—C19—H19A	108.5
C12—N13—C13	105.99 (19)	C18—C19—H19B	108.5
C12—N13—Cu1	122.46 (16)	C1A—C19—H19B	108.5
C13—N13—Cu1	131.53 (16)	H19A—C19—H19B	107.5
C22—N21—C21	107.20 (19)	C1B—C1A—C19	112.2 (2)
C22—N21—C28	127.3 (2)	C1B—C1A—H1A1	109.2
C21—N21—C28	125.5 (2)	C19—C1A—H1A1	109.2
C22—N23—C23	106.50 (19)	C1B—C1A—H1A2	109.2
C22—N23—Cu1	125.43 (15)	C19—C1A—H1A2	109.2
C23—N23—Cu1	127.91 (16)	H1A1—C1A—H1A2	107.9
C22—C1—C12	110.63 (19)	C1A—C1B—H1B1	109.5
C22—C1—C2	110.39 (19)	C1A—C1B—H1B2	109.5
C12—C1—C2	107.92 (18)	H1B1—C1B—H1B2	109.5
C22—C1—H1	109.3	C1A—C1B—H1B3	109.5
C12—C1—H1	109.3	H1B1—C1B—H1B3	109.5
C2—C1—H1	109.3	H1B2—C1B—H1B3	109.5
C3—C2—C1	114.4 (2)	N21—C21—C27	132.1 (2)
C3—C2—H2A	108.7	N21—C21—C23	106.0 (2)
C1—C2—H2A	108.7	C27—C21—C23	121.9 (2)
C3—C2—H2B	108.7	N23—C22—N21	111.9 (2)
C1—C2—H2B	108.7	N23—C22—C1	122.2 (2)
H2A—C2—H2B	107.6	N21—C22—C1	125.8 (2)
C2—C3—C4	112.0 (2)	N23—C23—C24	130.3 (2)
C2—C3—H3A	109.2	N23—C23—C21	108.4 (2)
C4—C3—H3A	109.2	C24—C23—C21	121.3 (2)
C2—C3—H3B	109.2	C25—C24—C23	117.0 (2)
C4—C3—H3B	109.2	C25—C24—H24	121.5
H3A—C3—H3B	107.9	C23—C24—H24	121.5
C5—C4—C3	114.3 (2)	C24—C25—C26	121.3 (2)
C5—C4—H4A	108.7	C24—C25—H25	119.3
C3—C4—H4A	108.7	C26—C25—H25	119.3
C5—C4—H4B	108.7	C27—C26—C25	122.2 (2)
C3—C4—H4B	108.7	C27—C26—H26	118.9
H4A—C4—H4B	107.6	C25—C26—H26	118.9
C4—C5—H5A	109.5	C26—C27—C21	116.2 (2)
C4—C5—H5B	109.5	C26—C27—H27	121.9
H5A—C5—H5B	109.5	C21—C27—H27	121.9
C4—C5—H5C	109.5	C28—C29—C2A	115.0 (2)
H5A—C5—H5C	109.5	C28—C29—H29A	108.5
H5B—C5—H5C	109.5	C2A—C29—H29A	108.5
C17—C11—N11	131.2 (2)	C28—C29—H29B	108.5
C17—C11—C13	122.6 (2)	C2A—C29—H29B	108.5
N11—C11—C13	106.2 (2)	H29A—C29—H29B	107.5
N13—C12—N11	112.1 (2)	N21—C28—C29	112.9 (2)
N13—C12—C1	124.0 (2)	N21—C28—H28A	109.0
N11—C12—C1	123.6 (2)	C29—C28—H28A	109.0
C14—C13—C11	120.3 (2)	N21—C28—H28B	109.0
C14—C13—N13	131.2 (2)	C29—C28—H28B	109.0
C11—C13—N13	108.5 (2)	H28A—C28—H28B	107.8
C15—C14—C13	117.2 (2)	C29—C2A—C2B	112.2 (2)
C15—C14—H14	121.4	C29—C2A—H2A1	109.2
C13—C14—H14	121.4	C2B—C2A—H2A1	109.2
C14—C15—C16	121.8 (2)	C29—C2A—H2A2	109.2
C14—C15—H15	119.1	C2B—C2A—H2A2	109.2
C16—C15—H15	119.1	H2A1—C2A—H2A2	107.9
C17—C16—C15	121.9 (2)	C2A—C2B—H2B1	109.5
C17—C16—H16	119.1	C2A—C2B—H2B2	109.5
C15—C16—H16	119.1	H2B1—C2B—H2B2	109.5
C16—C17—C11	116.3 (2)	C2A—C2B—H2B3	109.5
C16—C17—H17	121.9	H2B1—C2B—H2B3	109.5
C11—C17—H17	121.9	H2B2—C2B—H2B3	109.5
			
N23—Cu1—N13—C12	−27.22 (18)	C14—C15—C16—C17	0.0 (4)
Br1—Cu1—N13—C12	−160.30 (16)	C15—C16—C17—C11	0.1 (4)
Br2—Cu1—N13—C12	75.19 (19)	N11—C11—C17—C16	−176.7 (3)
N23—Cu1—N13—C13	154.6 (2)	C13—C11—C17—C16	−0.1 (4)
Br1—Cu1—N13—C13	21.6 (2)	C12—N11—C18—C19	91.0 (3)
Br2—Cu1—N13—C13	−102.9 (2)	C11—N11—C18—C19	−91.9 (3)
N13—Cu1—N23—C22	26.70 (19)	N11—C18—C19—C1A	70.4 (3)
Br1—Cu1—N23—C22	139.59 (16)	C18—C19—C1A—C1B	−178.6 (2)
Br2—Cu1—N23—C22	−108.60 (18)	C22—N21—C21—C27	178.3 (3)
N13—Cu1—N23—C23	−147.9 (2)	C28—N21—C21—C27	−1.4 (4)
Br1—Cu1—N23—C23	−35.1 (2)	C22—N21—C21—C23	−1.1 (3)
Br2—Cu1—N23—C23	76.75 (19)	C28—N21—C21—C23	179.1 (2)
C22—C1—C2—C3	56.9 (3)	C23—N23—C22—N21	−0.7 (3)
C12—C1—C2—C3	178.0 (2)	Cu1—N23—C22—N21	−176.26 (15)
C1—C2—C3—C4	−168.1 (2)	C23—N23—C22—C1	−177.4 (2)
C2—C3—C4—C5	−56.2 (3)	Cu1—N23—C22—C1	7.0 (3)
C12—N11—C11—C17	177.0 (3)	C21—N21—C22—N23	1.1 (3)
C18—N11—C11—C17	−0.6 (4)	C28—N21—C22—N23	−179.1 (2)
C12—N11—C11—C13	−0.1 (3)	C21—N21—C22—C1	177.7 (2)
C18—N11—C11—C13	−177.6 (2)	C28—N21—C22—C1	−2.5 (4)
C13—N13—C12—N11	−0.2 (3)	C12—C1—C22—N23	−47.6 (3)
Cu1—N13—C12—N11	−178.77 (15)	C2—C1—C22—N23	71.8 (3)
C13—N13—C12—C1	173.4 (2)	C12—C1—C22—N21	136.1 (2)
Cu1—N13—C12—C1	−5.2 (3)	C2—C1—C22—N21	−104.5 (3)
C11—N11—C12—N13	0.2 (3)	C22—N23—C23—C24	−179.5 (2)
C18—N11—C12—N13	177.7 (2)	Cu1—N23—C23—C24	−4.1 (4)
C11—N11—C12—C1	−173.5 (2)	C22—N23—C23—C21	−0.1 (3)
C18—N11—C12—C1	4.0 (4)	Cu1—N23—C23—C21	175.39 (16)
C22—C1—C12—N13	46.7 (3)	N21—C21—C23—N23	0.7 (3)
C2—C1—C12—N13	−74.2 (3)	C27—C21—C23—N23	−178.8 (2)
C22—C1—C12—N11	−140.4 (2)	N21—C21—C23—C24	−179.7 (2)
C2—C1—C12—N11	98.7 (3)	C27—C21—C23—C24	0.7 (4)
C17—C11—C13—C14	−0.1 (4)	N23—C23—C24—C25	178.5 (2)
N11—C11—C13—C14	177.3 (2)	C21—C23—C24—C25	−1.0 (4)
C17—C11—C13—N13	−177.5 (2)	C23—C24—C25—C26	0.3 (4)
N11—C11—C13—N13	−0.1 (3)	C24—C25—C26—C27	0.5 (4)
C12—N13—C13—C14	−176.8 (2)	C25—C26—C27—C21	−0.7 (4)
Cu1—N13—C13—C14	1.6 (4)	N21—C21—C27—C26	−179.3 (2)
C12—N13—C13—C11	0.2 (3)	C23—C21—C27—C26	0.1 (4)
Cu1—N13—C13—C11	178.54 (16)	C22—N21—C28—C29	−83.8 (3)
C11—C13—C14—C15	0.2 (3)	C21—N21—C28—C29	95.9 (3)
N13—C13—C14—C15	176.9 (2)	C2A—C29—C28—N21	−61.6 (3)
C13—C14—C15—C16	−0.2 (4)	C28—C29—C2A—C2B	−179.3 (2)

### Hydrogen-bond geometry (Å, °)

**Table:**

Cg1 is the centroid of the N11/C11/C13/N13/C12 ring.

**Table:**

*D*—H···*A*	*D*—H	H···*A*	*D*···*A*	*D*—H···*A*
C14—H14···Br1	0.95	2.79	3.551 (3)	138
C17—H17···Br2^i^	0.95	2.90	3.606 (3)	132
C18—H18A···Br1^ii^	0.99	2.86	3.741 (3)	148
C5—H5B···Cg1^iii^	0.98	2.87	3.631 (3)	135
C2B—H2B1···Cg1^iv^	0.98	2.82	3.777 (3)	165

Symmetry codes: (i) *x*+1/2, −*y*+1/2, *z*−1/2; (ii) −*x*+1/2, *y*+1/2, −*z*+1/2; (iii) −*x*+1/2, *y*+1/2, −*z*+1/2; (iv) *x*+1/2, −*y*+1/2, *z*+1/2.
